# Supramolecular Complexation of Niclosamide and Nafamostat: Improved Physicochemical Profile and Enhanced Anticancer Effect

**DOI:** 10.3390/ph19081277

**Published:** 2026-08-13

**Authors:** Se-Eun Byeon, Rengarajan Baskaran, Young-Joon Park

**Affiliations:** 1 Research Center, IMD Pharm Inc., 17 Daehak 4-ro, Yeongtong-gu, Suwon 16226, Gyeonggi-do, Republic of Korea; 2 College of Pharmacy, Ajou University, 206 World Cup-Ro, Yeongtong-gu, Suwon 16499, Gyeonggi-do, Republic of Korea

**Keywords:** niclosamide, nafamostat mesylate, supramolecular crystalline phase, bioavailability, anticancer

## Abstract

**Background/Objective**: Niclosamide exhibits considerable potential for the treatment of drug-resistant cancers. However, its poor aqueous solubility substantially limits its oral bioavailability and therapeutic efficacy. To overcome this limitation, we developed a novel pharmaceutical supramolecular crystalline phase comprising niclosamide and nafamostat. **Methods**: In this study, we developed a niclosamide–nafamostat pharmaceutical supramolecular crystalline phase using a conventional solvent evaporation technique. Comprehensive solid-state characterization was carried out using X-ray powder diffraction (XRD), Fourier-transform infrared spectroscopy (FTIR), and differential scanning calorimetry (DSC). In vitro drug transport kinetics and barrier transport efficiency were evaluated across artificial transpermeable membranes using a diffusion cell setup. In vitro anticancer efficacy was systematically screened against human lung, breast, and pancreatic cancer cell lines. Finally, in vivo translation was established using female NOD/SCID mice tumor-bearing animal models; therapeutic efficacy and total tumor burden were evaluated. **Results**: Niclosamide–nafamostat cocrystal (NNC) was found to form a distinct crystalline phase, with characterization results supporting a unique crystal lattice stabilized through strong intermolecular hydrogen-bonding interactions and exhibiting high thermal purity. Crucially, the supramolecular crystalline phase considerably enhances membrane transport, yielding superior cumulative permeation and enhanced apparent permeability (Papp) values compared to those of pure niclosamide. In vitro evaluation across multiple cancer cell lines demonstrated a tenfold increase in antiproliferative potency compared to the parent compounds. Furthermore, in vivo studies revealed a twofold increase in tumor growth inhibition and a tenfold reduction in total tumor burden (*p* < 0.05). **Conclusions**: These findings demonstrate that the NNC supramolecular crystalline phase platform successfully optimizes membrane permeability and antitumor efficacy, highlighting its potential for treating advanced cancers.

## 1. Introduction

Cocrystal drug development is a rapidly evolving area of pharmaceutical research aimed at overcoming the physicochemical limitations that often compromise the clinical performance of promising therapeutic agents [1,2]. This approach is particularly relevant in oncology, where cancer remains one of the leading causes of death worldwide [3,4]. Pharmaceutical cocrystallization has gained considerable attention as an effective crystal-engineering strategy to optimize the biopharmaceutical properties of active pharmaceutical ingredients (APIs), particularly those classified as biopharmaceutics classification system classes II and IV [5,6]. By modulating noncovalent intermolecular interactions within the crystal lattice without altering the covalent structure or intrinsic pharmacological activity of the parent APIs, a pharmaceutical supramolecular crystalline phase can considerably enhance aqueous solubility, dissolution rates, solid-state stability, and control over polymorphic behavior. The specific physicochemical improvements are of pharmacological importance as they directly dictate the drugs’ in vivo dissolution kinetics, maximize systemic bioavailability, and optimize therapeutic efficacy during clinical translation [7,8,9,10]. In parallel, the repurposing and reformulation of approved drugs with newly recognized anticancer potential have emerged as attractive and cost-effective approaches for accelerating therapeutic innovation [11,12,13,14].

Niclosamide, an FDA-approved anthelmintic agent, has gained substantial attention as a repurposed anticancer drug [11,12]. Numerous studies have demonstrated its ability to overcome chemotherapeutic resistance across diverse tumor types by modulating key oncogenic signaling pathways, inducing apoptosis, and suppressing cancer stem cell-like phenotypes [13,15]. In addition, niclosamide has been shown to enhance the efficacy of immunotherapeutic strategies, including immune checkpoint inhibitors, by remodeling the immunosuppressive tumor microenvironment and attenuating mechanisms of immune evasion [16]. Despite this therapeutic promise, niclosamide suffers from exceptionally poor aqueous solubility and limited systemic exposure [8]. These unfavorable pharmacokinetic characteristics represent major barriers to its clinical translation in oncology [17,18,19]. Nafamostat mesylate (nafamostat) is a synthetic serine protease inhibitor that is clinically used for treating acute pancreatitis, acute respiratory distress syndrome, and disseminated intravascular coagulation. Beyond its established anticoagulant and anti-inflammatory properties, nafamostat has demonstrated promising anticancer activity, thereby expanding its potential therapeutic utility in treating lung cancer [20]. However, its clinical application is heavily constrained by rapid in vivo hydrolysis, poor cellular permeability, and a short systemic half-life, which complicate sustained systemic treatment and necessitate stable delivery systems [20,21].

Pharmaceutical cocrystallization offers an elegant approach for simultaneously addressing the limitations of both compounds [22]. A supramolecular crystalline phase comprises two or more distinct molecules held together in a single crystalline phase through noncovalent interactions, including hydrogen bonding, van der Waals forces, and π–π stacking interactions. Such systems can be prepared through various scalable and industrially relevant methods, including solution-based techniques (solvent evaporation, cooling crystallization, and spray drying) and mechanochemical approaches (liquid-assisted grinding and hot-melt extrusion) [23]. Crucially, dual-API combines two pharmacologically active compounds into a single solid-state form, thereby providing opportunities to optimize dissolution behavior, optimize physicochemical performance, and potentially achieve therapeutic effects while minimizing dose-dependent toxicity [9,24,25].

Given the complementary anticancer, antiviral, and anti-inflammatory activities of niclosamide and nafamostat, the design of a dual-API incorporating both agents presents a compelling strategy for overcoming their respective limitations in solubility, permeability, and bioavailability. In this study, we designed and prepared a novel NNC to modulate intermolecular packing arrangements and optimize critical solid-state properties. Structural characterization was combined with in vitro using different cancer cell lines and in vivo evaluations using an orthotopic PANC-1 pancreatic cancer model to investigate whether the supramolecular crystalline phase exhibits superior therapeutic performance compared to the individual APIs and their corresponding physical mixtures. Ultimately, this study aims to establish an innovative solid-state drug-delivery platform capable of enhancing therapeutic efficacy while addressing key pharmacokinetic challenges associated with both compounds.

## 2. Results

### 2.1. Fourier-Transform Infrared Spectroscopy

**Figure 1:**
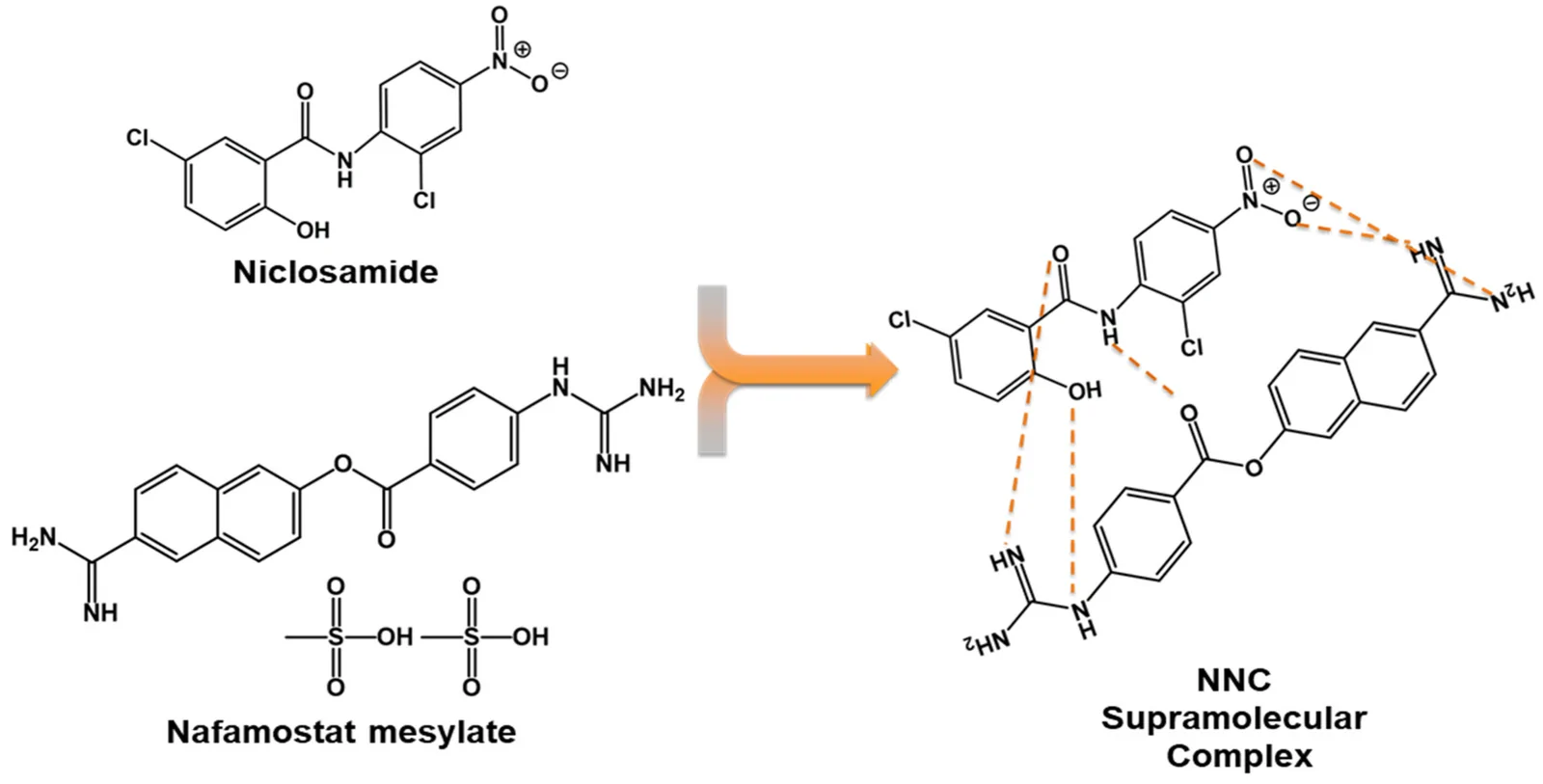
Proposed schematic representation of the intermolecular hydrogen-bonding arrangement within the niclosamide–nafamostat supramolecular crystalline phase. Orange dashed lines indicate hypothesized supramolecular interactions between complementary hydrogen-bond donor and acceptor groups.

**Figure 2:**
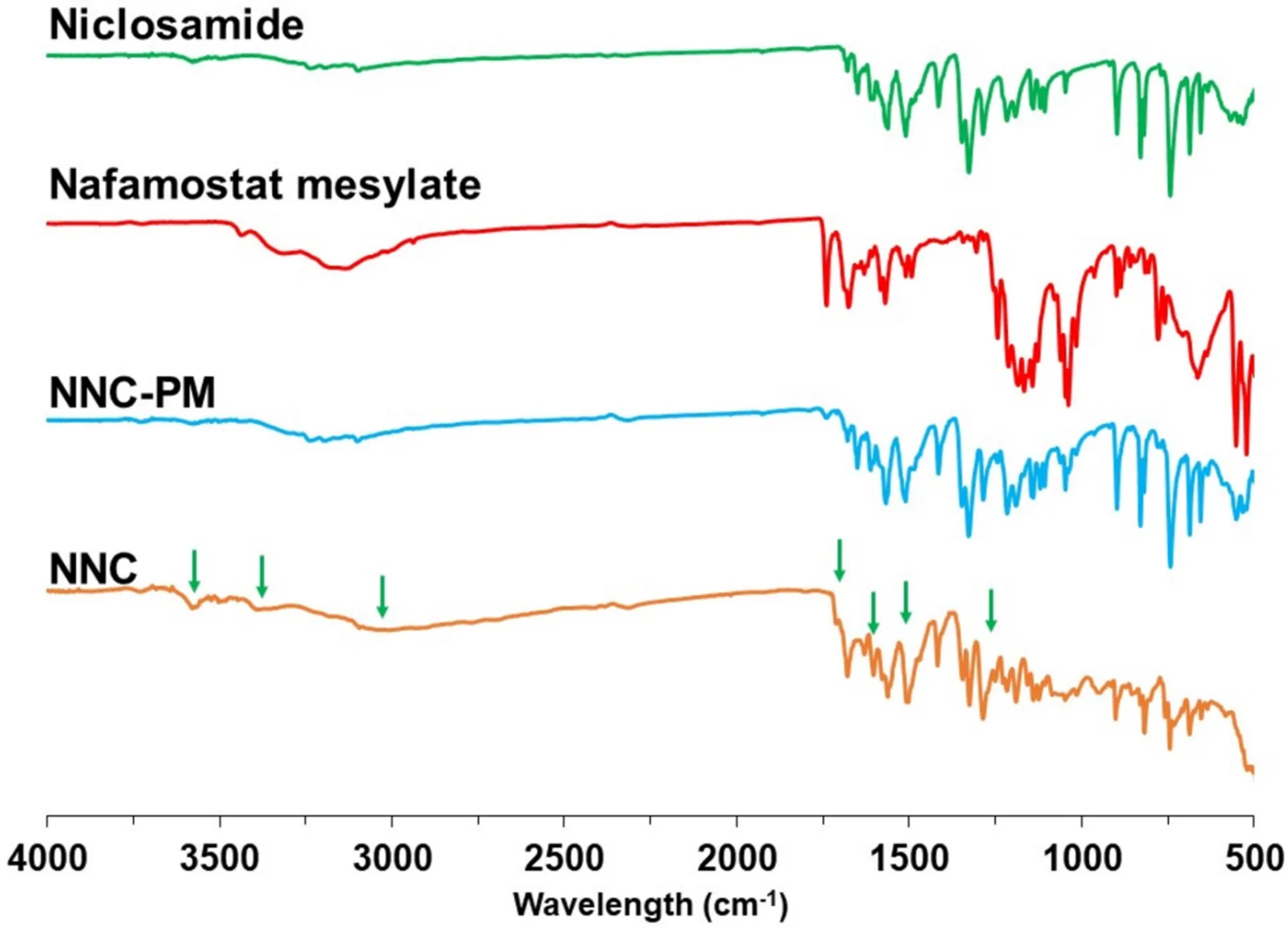
FTIR spectra of niclosamide, nafamostat, the physical mixture, and the NNC supramolecular crystalline phase were evaluated for identifying possible intermolecular interactions. The physical mixture exhibited the characteristic peaks of both drugs without any notable changes, suggesting minimal interactions between them. By contrast, the NNC spectrum exhibited peak shifting and the appearance of new absorption bands of O–H and N–H stretching compared with the physical mixture. Green arrows highlight critical spectral shifts, peak broadening, and the emergence of new absorption bands unique to the NNC phase. These spectral changes indicate the formation of intermolecular interactions, such as hydrogen bonding, resulting in successful supramolecular crystalline phase formation.

The NNC supramolecular crystalline phase was prepared by a conventional solvent evaporation method, as shown in Figure 1. The resulting crystal lattice is proposed to be stabilized by non-covalent intermolecular interactions, including possible hydrogen bonding between niclosamide and nafamostat molecules. The formation of NNC was investigated using Fourier-transform infrared spectroscopy (FTIR) for structural confirmation of the supramolecular crystalline phase (Figure 2). The FTIR spectra revealed notable shifts in characteristic vibrational frequencies, indicating the formation of hydrogen-bonding interactions within the supramolecular crystalline phase structure. Although the physical mixture merely combines the individual drugs without changing them, the NNC showed major structural shifts that indicate the formation of a distinct molecular environment.

A great vibrant change occurred in the high-frequency range of 3600–3000 cm^−1^, where the individual O–H and N–H peaks broadened, merged, and considerably shifted downward to 3021 cm^−1^. The dramatic peak shift suggests the highly reactive phenolic O–H group of niclosamide. Niclosamide acts as a primary hydrogen bond donor to nafamostat. Furthermore, the amide I band associated with C=O stretching vibrations shifted to 1697 cm^−1^, indicating alterations in the electronic environment of the carbonyl group and supporting its participation in intermolecular interactions. Further evidence of supramolecular crystalline phase formation was observed in the fingerprint region, where a new absorption band appeared at 1157 cm^−1^, accompanied by shifts in the S=O and C–O stretching vibrations toward approximately 1250 cm^−1^. These spectral changes suggest a reorganization of the local molecular environment and are consistent with modifications to the crystal packing arrangement. Moreover, additional shifts in the 902–889 cm^−1^ region provide further evidence of intermolecular interactions between niclosamide and nafamostat within the supramolecular crystalline phase structure. Collectively, the observed spectral changes support the successful formation of a stable niclosamide–nafamostat supramolecular crystalline phase characterized (Table S1) by a distinct hydrogen-bonding network and a modified crystal lattice relative to the parent compounds.

### 2.2. Crystallinity and Phase Transition Evaluation

**Figure 3:**
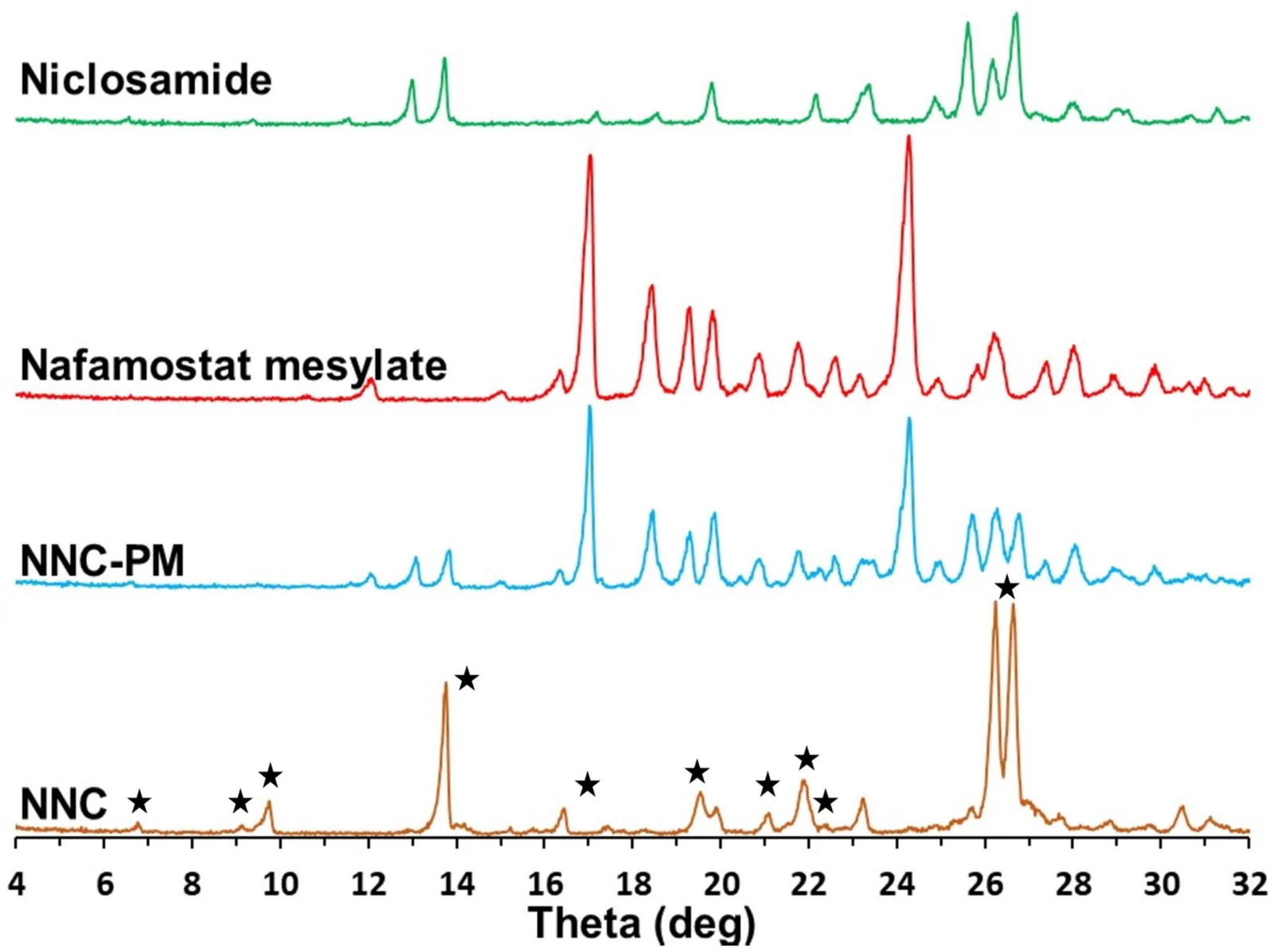
Comparative PXRD patterns of niclosamide, nafamostat, their physical mixture (PM), and the supramolecular crystalline phase of NNC. The PM pattern displays an exact superposition of the individual raw materials, indicating that no structural changes occur upon mechanical mixing. By contrast, the NNC supramolecular crystalline phase diffractogram exhibits the disappearance of parent peaks and the emergence of distinct new crystalline peaks marked with stars. This distinct pattern shows the successful formation of a crystalline phase structure.

Powder X-ray diffraction analysis supports the successful formation of a distinct crystalline phase for the NNC, marking a structural transformation from starting materials (Figure 3). The physical mixture merely superimposed the crystalline fingerprints of both niclosamide and nafamostat. It maintained the sharp characteristic peaks of niclosamide near 13°, 14°, and 25–27° cluster, right next to the big nafamostat peaks at 17° and 24°. This shows that simply mixing the niclosamide and nafamostat together without changing their basic structures. However, the PXRD pattern of NNC showed the complete disappearance of these original drugs. The total disappearance evidences that the old crystal structures broke down with no unreacted components. Instead, a unique crystal structure was evidenced by sharp, highly intense new peaks at 9.8°, 13.8°, and a distinct double peak at 26.2° and 26.6°. These new peaks demonstrate that the intermolecular hydrogen bonds successfully locked both drugs into a highly organized, stable, and clear supramolecular crystalline phase.

### 2.3. Thermal Behavior Assessment

**Figure 4:**
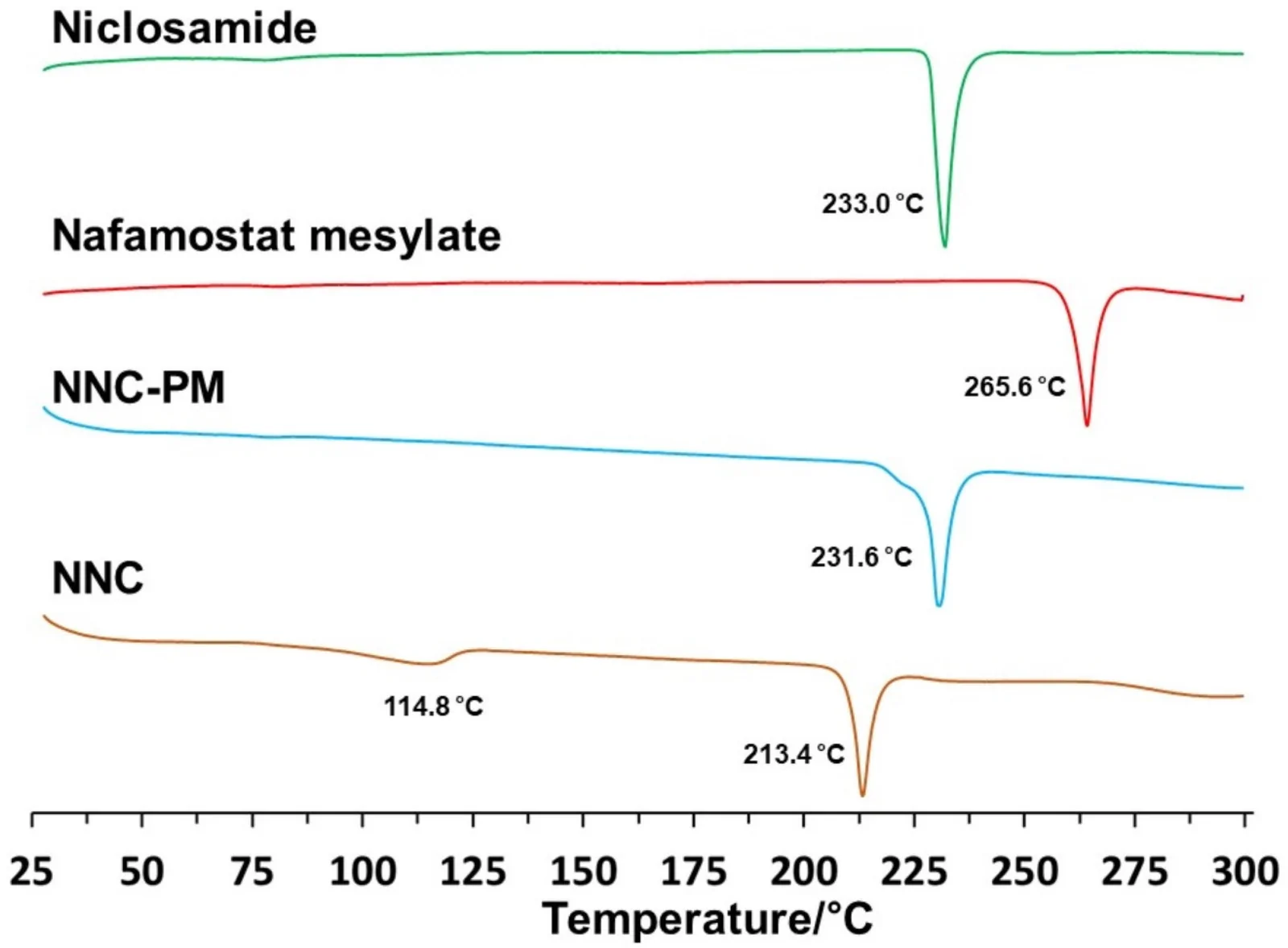
Thermal analysis curves for niclosamide, nafamostat, the physical mixture (PM), and the NNC. Pure niclosamide and nafamostat exhibited characteristic endothermic peaks at 233.0 °C and 265.6 °C, respectively. The physical mixture showed a peak at 231.6 °C, indicating the retention of the drug’s crystalline nature. By contrast, the NNC displayed shifted peaks at 213.4 °C and 114.8 °C. These distinct thermal shifts are strongly consistent with the presence of intermolecular interactions and support the proposed formation of a new supramolecular crystalline phase, demonstrating intermolecular interactions and successful supramolecular crystalline phase formation.

Differential scanning calorimetry (DSC) was employed to investigate the thermal behavior of NNC and to provide evidence for forming a new solid phase (Figure 4). Pure niclosamide exhibited a sharp endothermic melting peak at 233.0 °C, whereas nafamostat displayed a distinct melting endotherm at 265.6 °C. The physical mixture showed a principal melting event at 231.6 °C, indicating that simple blending of the two compounds did not result in the formation of a new crystalline phase. By contrast, the DSC thermogram of the NNC revealed the disappearance of the characteristic melting peaks associated with the parent compounds and the emergence of a new, well-defined endothermic peak at 213.4 °C. The appearance of a single melting event at a temperature distinct from those of the individual components is consistent with the formation of a new crystalline phase. The optimized scale-up process demonstrated high efficiency and reproducibility, providing a mean isolated synthesis yield of 75.76 ± 5.35% across four independent batches. The resulting samples were characterized by DSC to evaluate the consistency of their thermal properties (Figure S1). Furthermore, the absence of the characteristic thermal transitions of niclosamide and nafamostat strongly suggests that the supramolecular crystalline phase predominates within the analyzed sample. The observed thermal behavior supports the successful formation of a niclosamide–nafamostat supramolecular crystalline phase possessing physicochemical properties distinct from those of the parent compounds. The unique melting profile is indicative of altered intermolecular interactions and supramolecular crystalline phase packing within the supramolecular crystalline phase lattice, consistent with the establishment of a new solid-state form.

### 2.4. Equilibrium Solubility Studies

The equilibrium solubility of the NNC supramolecular crystalline phase was evaluated alongside pure niclosamide across a physiologically relevant pH range (Figure S2). NNC demonstrated consistently higher aqueous solubility than pure niclosamide under all tested conditions. Specifically, in distilled water (DW), the solubility increased from 0.7 ± 0.45 g/L for niclosamide to 1.78 ± 0.39 g/L for NNC. This enhancement trend was maintained across the buffer media, with NNC reaching maximum solubility values of 4.37 ± 0.67 g/L at pH 4.0 and 4.30 ± 0.46 g/L at pH 6.8, compared to 2.53 ± 0.82 g/L and 3.10 ± 0.36 g/L for the parent drug, respectively. These findings indicate that the formation of the supramolecular crystalline phase effectively alters the crystal lattice energy, significantly improving the apparent aqueous solubility of niclosamide regardless of environmental acidity.

### 2.5. Ambient Stability Study

Ambient stability testing revealed that niclosamide, nafamostat, and NNC remained highly stable over a 1-month storage period under standard controlled conditions (25 ± 2 °C/60 ± 5% RH). All three test groups retained nearly 100% of their initial active concentrations (Figure S3), demonstrating negligible chemical degradation. Quantitative chemical assay analysis showed recovery values of 99.17 ± 0.91% for niclosamide, 98.87 ± 0.99% for nafamostat, and 99.10 ± 0.82% for NNC.

### 2.6. In Vitro Drug Permeability Study

**Figure 5:**
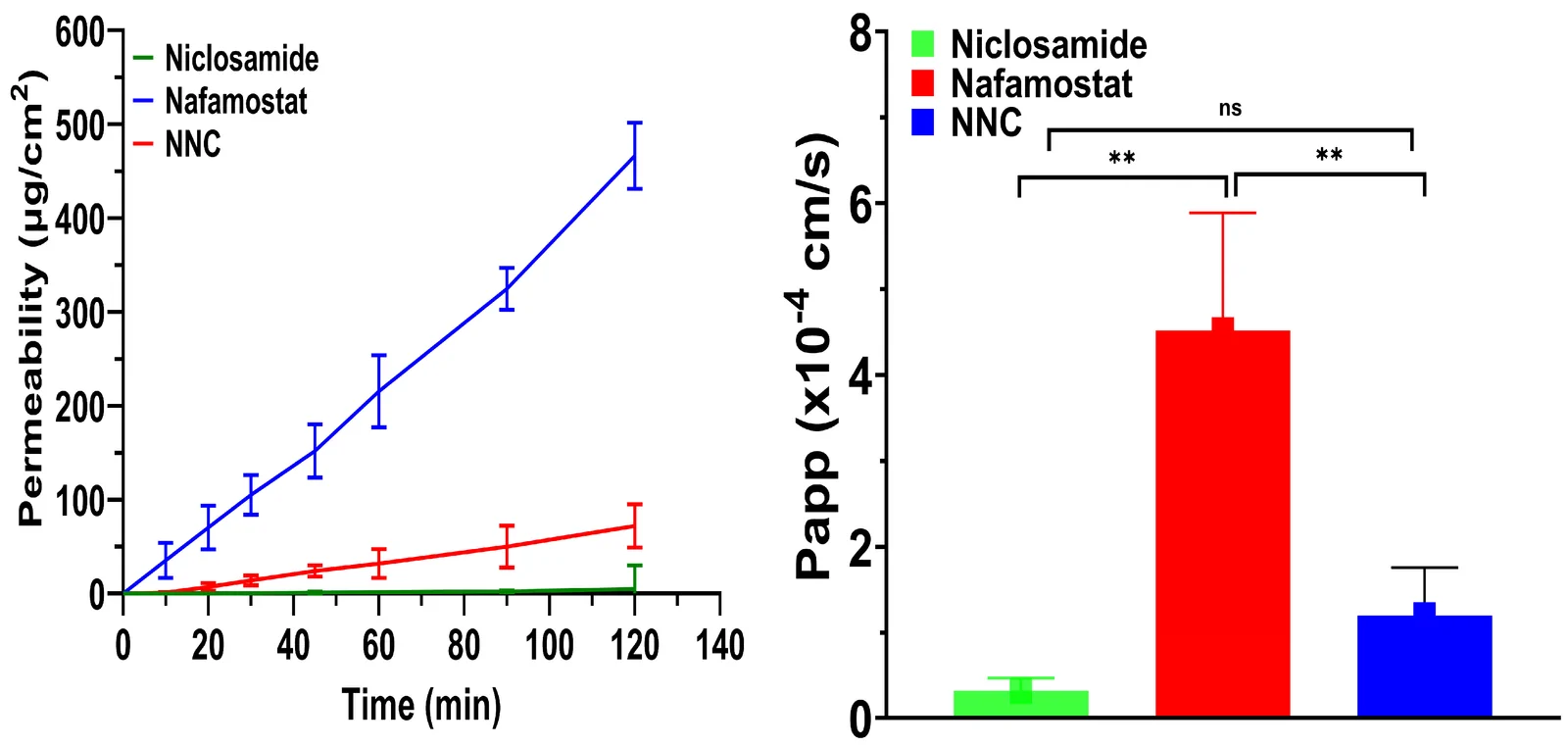
Cumulative in vitro permeability–time profiles (left) and permeability rate (Papp) of pure niclosamide, nafamostat, and the NNC supramolecular crystalline phase over a 120-min monitoring period. Pure niclosamide exhibits negligible permeability owing to its highly hydrophobic nature, whereas pure nafamostat exhibits high permeability. Notably, the NNC supramolecular crystalline phase considerably enhanced the cumulative permeation of niclosamide, highlighting the effectiveness of structural crystallization in improving the transdermal transport and dissolution behavior of the poorly soluble parent drug. Longitudinal profiles were evaluated using a two-way repeated-measures ANOVA, while statistical differences across Papp values were determined via a one-way Analysis of Variance (ANOVA) followed by Tukey’s post hoc multiple comparisons test in GraphPad Prism. Highly significant variations are indicated by asterisks (** *p* < 0.05), while non-significant differences are denoted as ns (*p* > 0.05).

The cumulative in vitro membrane permeation profiles and calculated apparent permeability coefficients (Papp) for pure niclosamide, nafamostat mesylate, and NNC over 120 min are shown in Figure 5. Pure nafamostat mesylate crossed the membrane rapidly, showing a high-flux profile that peaked at a cumulative transport of 466.8 ± 34.1 µg/cm^2^ and a high permeability coefficient (Papp = 4.43 ± 1.33 × 10^−4^ cm/s). In contrast, pure niclosamide showed limited membrane transport, trailing flat along the baseline due to poor solubility and dissolution rate (12.5 ± 5.1 µg/cm^2^; Papp = 0.31 ± 0.12 × 10^−4^ cm/s). Interestingly, tracking the kinetics of the NNC phase revealed a distinctly optimized, intermediate behavior that reached 72.3 ± 23.4 µg/cm^2^. This shift represents a highly significant fold increase in the apparent permeability coefficient (Papp = 1.12 ± 0.55 × 10^−4^ cm/s) compared to pure niclosamide. This enhanced transport behavior can be directly attributed to modifications in the solid-state properties and dissolution characteristics resulting from NNC. Together, these findings demonstrated that the NNC represents a highly promising approach for improving the permeability performance of niclosamide.

### 2.7. In Vitro Anticancer Activity

**Figure 6:**
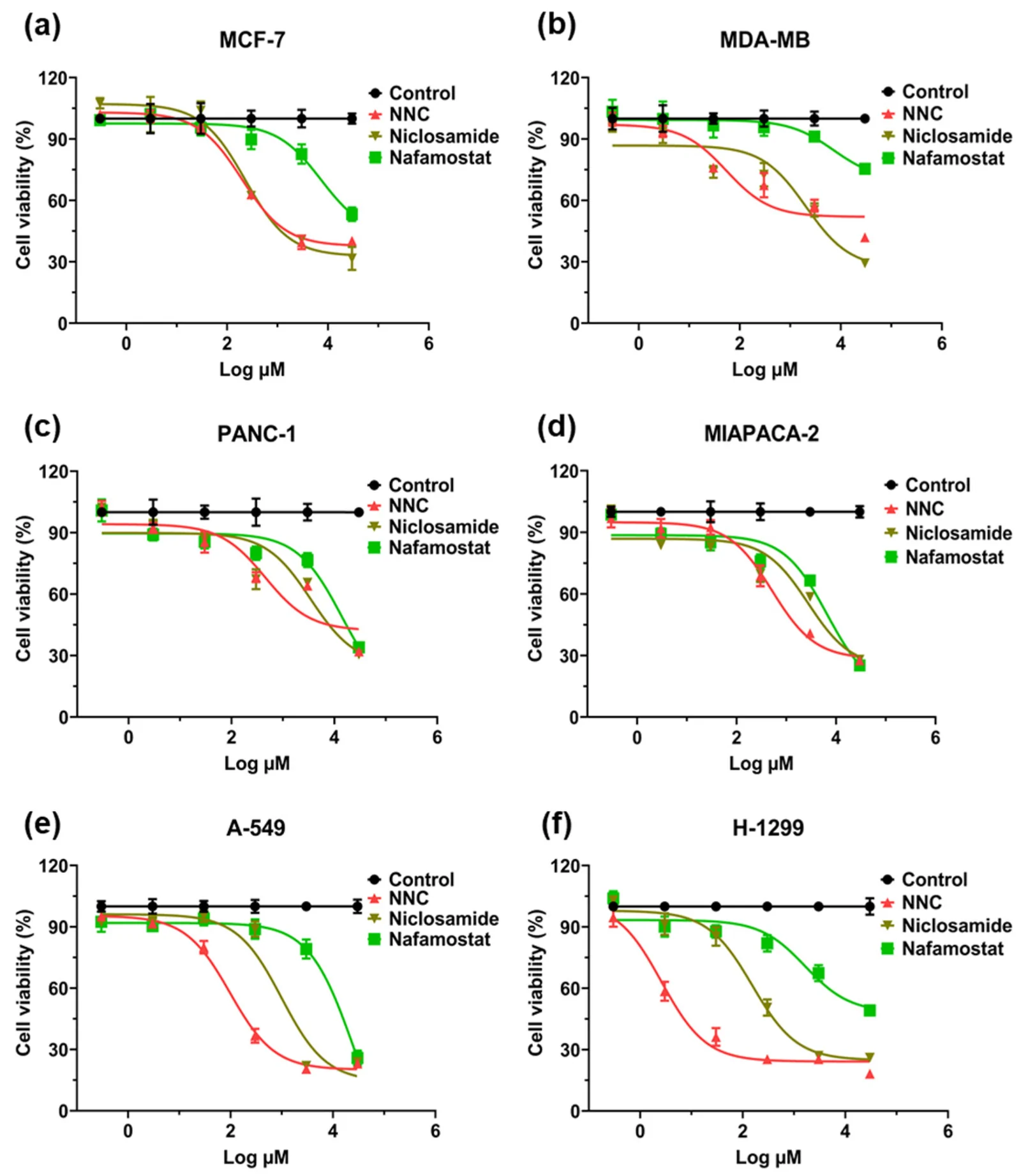
Anticancer activity of niclosamide, nafamostat, and NNC against different cancer cell lines, including (**a**,**b**) breast cancer ((**a**). MCF-7, (**b**). MDA-MB), (**c**,**d**) pancreatic cancer ((**c**). PANC-1, (**d**). MIAPACA-2), and (**e**,**f**) lung cancer cells ((**e**). A-549, (**f**). H-1299). Cell viability was evaluated after 48 h of incubation at 37 °C in a humidified atmosphere containing 5% CO_2_. The dose–response curves demonstrate that the NNC supramolecular crystalline phase formulation (red) exhibits considerably enhanced or comparable cytotoxicity relative to pure niclosamide (black) and superior efficacy over nafamostat alone (green) across all tested lines, particularly highlighting a pronounced potency shift in the lung cancer lines (**e**,**f**). Data points represent the mean values ± standard deviation (SD) from independent replicates.

The anticancer activity of NNC was evaluated across multiple cancer cell lines, including lung, breast, and pancreatic cancer models (Figure 6). Overall, NNC exhibited enhanced antiproliferative activity compared with the individual parent compounds, as reflected by its lower IC_50_ values in several cell lines. Among the tested models, the most pronounced activity was observed in lung cancer cells. In H-1299 cells, NNC achieved an IC_50_ value of 2.596 nM (95% Cl 0.623 to 4.18 nM), representing a substantial potency increase over niclosamide (IC_50_ = 153.2 nM) and nafamostat (IC_50_ = 1642 nM). Similarly, in A-549 cells (Table S3), NNC demonstrated potency with an IC_50_ of 97.28 nM compared to niclosamide (990.9 nM) and nafamostat (25,173 nM). By contrast, nafamostat alone demonstrated relatively limited antiproliferative activity across all the cell lines, with an IC_50_ value exceeding 6500 nm in five out of six models. Compared with the individual compounds, the enhanced activity of the NNC in lung cancer cell lines suggests that supramolecular crystalline phase formation is more beneficial in biological performance. However, the therapeutic advantage of the supramolecular crystalline phase was less pronounced in breast and pancreatic cancer cell lines, where NNC displayed moderate potency profiles, such as an IC_50_ of 196.4 nM in MCF-7 cells (Table S2) and 452.6 nM in PANC-1 cells (Table S3). Notably, in MDA-MB breast cancer cells, the NNC exhibited lower cytotoxicity than pure niclosamide at higher concentrations, yielding an IC_50_ of 54.07 nM compared to niclosamide 2042 nM under these specific assay conditions. The antiproliferative activity observed in lung cancer models may be associated with alterations in physicochemical properties resulting from supramolecular crystalline phase formation, including enhanced solubility and permeability. Collectively, these findings indicate that the dual-API supramolecular crystalline phase exhibits promising anticancer activity, particularly against lung cancer cell lines, and warrants further investigation as a potential therapeutic platform. Future testing against an equimolar physical mixture is needed for solubility advantages from baseline combination pharmacology.

### 2.8. In Vivo Antitumor Activity

**Figure 7:**
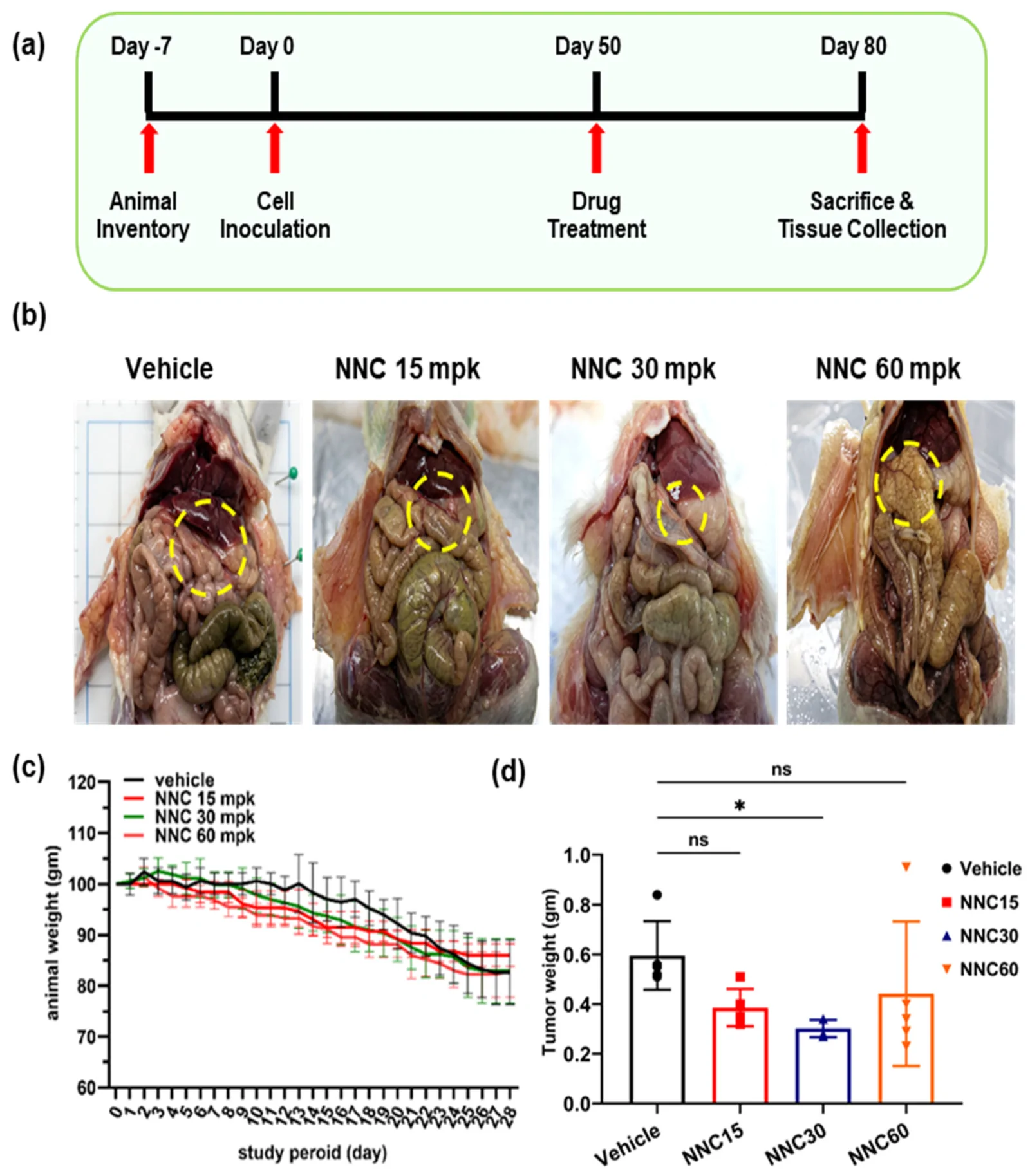
In vivo tumor growth suppression study following the intraperitoneal administration of NNC at different doses over 80 days in a tumor-bearing animal model. (**a**) Experimental timeline. (**b**) Representative images of vehicle and NNC-treated animals (yellow dashed circles delineate the primary tumor site). (**c**) Animal body weight changes during the study. (**d**) Tumor weight analysis. Animals treated with normal saline served as the vehicle control group, whereas NNC supramolecular crystalline phase formulations were administered at doses of 15, 30, and 60 mg/kg. No significant changes in body weight were observed throughout the treatment period, suggesting that NNC administration was generally well tolerated under the experimental conditions. Tumor growth remained suppressed in the 15 and 30 mg/kg treatment groups compared with the vehicle control, indicating substantial antitumor activity. By contrast, the 60 mg/kg group exhibited reduced therapeutic efficacy and increased tumor burden relative to the lower-dose treatment groups. One possible explanation is incomplete dissolution or suboptimal dispersion of the administered formulation at the higher dose; however, further investigation is required to confirm this hypothesis. Tumor burden decreased with increasing NNC dose up to 30 mg/kg relative to the vehicle-treated group. Statistical significance was defined as * *p* < 0.05, while non-significant differences are denoted as ns (*p* > 0.05).

To further assess the antitumor efficacy of NNC, in vivo studies were conducted using a murine pancreatic tumor model (Figure 7). Administration of NNC at doses of 15, 30, and 60 mg/kg resulted in varying degrees of tumor growth inhibition compared with the vehicle-treated control group. Treatment with 15 mg/kg NNC substantially reduced the average tumor weight, whereas the 30 mg/kg dose produced the most pronounced antitumor effect, resulting in a significant reduction in tumor burden. Interestingly, increasing the dose to 60 mg/kg did not lead to a further improvement in antitumor efficacy. Instead, the therapeutic response appeared to plateau or decrease relative to that observed at the intermediate dose. This nonlinear dose–response relationship may reflect factors affecting drug disposition, bioavailability, tumor penetration, or other pharmacokinetic and pharmacodynamic processes; however, additional studies are required to elucidate the underlying mechanism. Body weight monitoring throughout the treatment period revealed comparable trends across all experimental groups, suggesting that NNC administration was not associated with overt systemic toxicity under the conditions examined. Collectively, these findings demonstrate that the NNC supramolecular crystalline phase exhibits significant antitumor activity in vivo and highlight the importance of dose optimization for maximizing therapeutic efficacy.

### 2.9. In Vivo Tumor Burden Study

**Figure 8:**
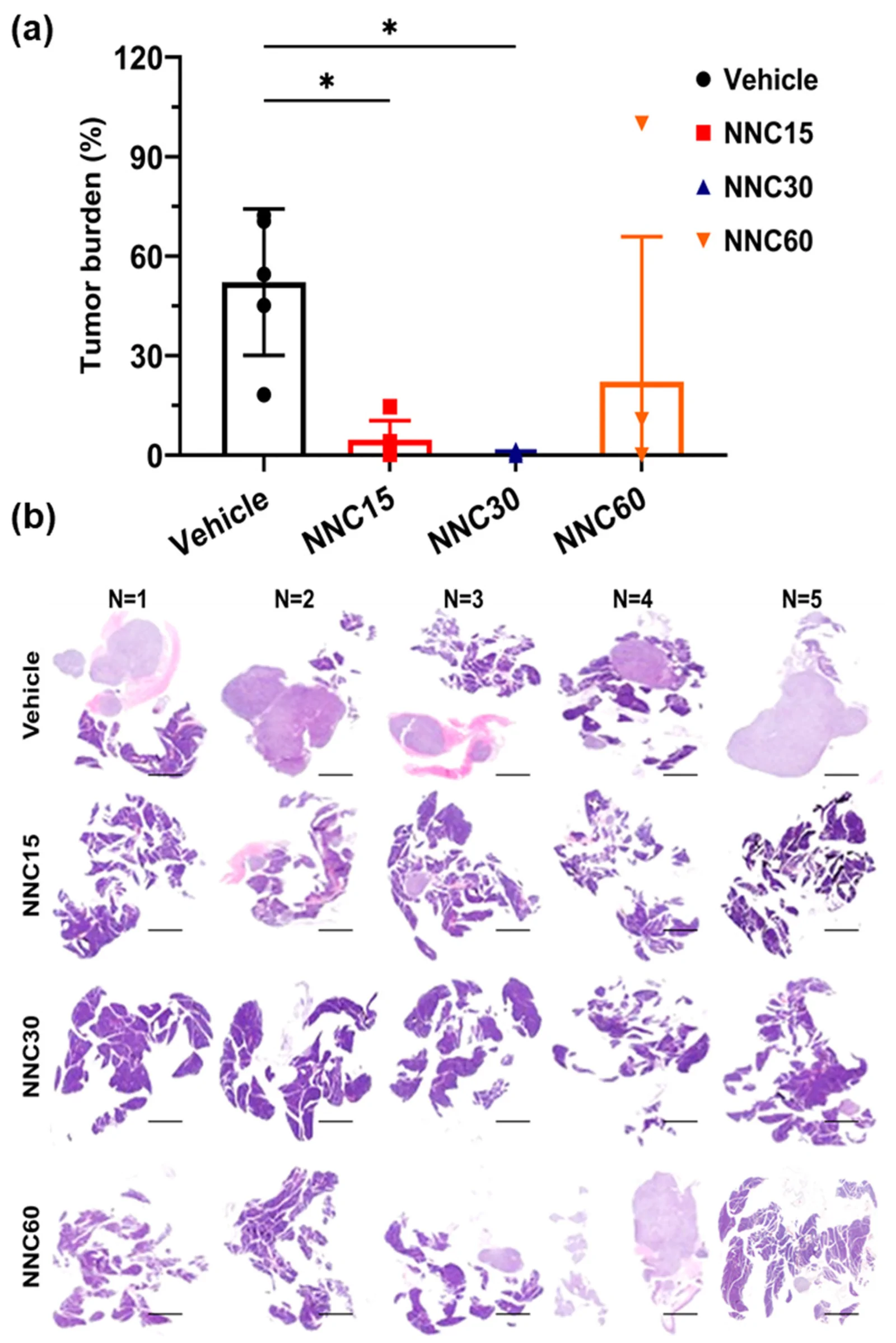
In vivo tumor burden study evaluating NNC at doses of 15, 30, and 60 mg/kg. (**a**) Quantification of tumor burden following treatment. (**b**) Representative images of sectioned and stained tumor tissues collected at the end of the experiment. The vehicle-treated group exhibited a higher tumor burden than the NNC-treated groups. Tumor burden progressively decreased with increasing NNC dose up to 30 mg/kg (Scale bar = 500 um). Statistical significance between the treatment and vehicle groups was determined via a one-way Analysis of Variance (ANOVA) followed by Tukey’s post hoc multiple comparisons test using GraphPad Prism software (version 9.5.1). Statistical significance was determined as * *p* < 0.05.

The tumor burden analysis further supported the in vivo antitumor activity of the prepared NNC formulation, as illustrated in Figure 8. Based on the absolute harvested weights, treatment with NNC at 15 mg/kg achieved a robust tenfold reduction in mean tumor mass compared to the vehicle control group. This therapeutic response reached its maximum in the 30 mg/kg cohort, which demonstrated a profound 120-fold reduction, indicating the maximum tumor clearance. This trend is primarily attributed to a maximum intestinal solubility ceiling combined with individual animal variance. These quantitative mass metrics correlate perfectly with the tissue sections from both the 15 and 30 mg/kg cohorts, which display drastically minimal tumor involvement compared to the extensive malignant tissue grids visible in the untreated controls. Furthermore, these potent antitumor responses at 15 and 30 mg/kg (Figure 8b) occurred without inducing any formulation-specific systemic toxicity; the relative body weight changes (%) remained closely parallel across all experimental groups, with the observed gradual decline reflecting progressive, tumor-associated cachexia characteristic of this aggressive orthotopic model. Interestingly, the relative mean tumor burden percentage for the 60 mg/kg cohort appeared higher on the graph (Figure 8a), resulting in an apparent nonlinear dose–response profile. Increasing dose is restricted by vehicle volume, which reached a local thermodynamic saturation point in the gastrointestinal tract, leading to incomplete in vivo dissolution and localized precipitation that limited further systemic drug absorption. However, this numerical shift was driven entirely by a single high-variance outlier animal (N = 4), whereas the absolute tissue weights for the remaining replicates remained highly suppressed, maintaining a substantial fortyfold (40-fold) absolute mass reduction. These findings demonstrate that the NNC complex exhibits remarkable in vivo efficacy, with the 30 mg/kg dose level yielding a potential therapeutic benefit.

Overall, the results demonstrate the successful formation of a novel NNC. Physicochemical characterization revealed the establishment of new intermolecular interactions, consistent with hydrogen-bond formation between the constituent molecules. X-ray diffraction analysis revealed the disappearance of characteristic diffraction peaks associated with the parent compounds and the emergence of new diffraction patterns, indicating the formation of a distinct crystalline phase. Thermal analysis further supported supramolecular crystalline phase formation through the appearance of a single melting event, consistent with the presence of a new solid-state form. The NNC considerably enhanced cumulative permeation and increased the Papp relative to pure niclosamide. Biological evaluation demonstrated promising anticancer activity across multiple cancer cell lines. Although reduced efficacy was observed at the highest dose tested in vivo, the underlying mechanism requires further investigation. Collectively, these findings establish a solid foundation for the continued development of this supramolecular crystalline phase platform as a potential strategy for improving anticancer drug delivery.

## 3. Discussion

The present study demonstrates that forming a novel NNC supramolecular crystalline phase fundamentally alters the solid-state properties of the parent drugs in a way that is consistent with true supramolecular crystalline phase formation rather than simple physical mixing. The combined evidence from FTIR, PXRD, and DSC results is consistent with the spectral shifts and new diffraction peaks, alongside the loss of characteristic reflections of the individual components. The simultaneous absence of parent X-ray reflections and the isolation of a singular, sharp thermal event collectively provide robust proof of macroscopic phase purity and structural homogeneity within the newly engineered supramolecular crystalline phase. The emergence of a new crystalline phase, while the single, well-defined melting endotherm indicates a uniform solid form with distinct intermolecular interactions. Taken together, these findings suggest that cocrystallization created a stable hydrogen-bonded lattice that reorganizes the crystal packing and provides a structural basis for the subsequent changes in physicochemical behavior. The apparent solubility of NNC across all tested pH conditions is consistent with alterations in the solid-state structure and intermolecular interactions relative to the parent drug. Further, NNC demonstrated excellent chemical integrity over a one-month screening period under standard ambient storage conditions (25 ± 2 °C/60 ± 5% RH); long-term phase behavior remains to be fully characterized. Future evaluations under accelerated stress conditions (40 ± 2 °C/75 ± 5% RH) are necessary to thoroughly monitor potential polymorphic transformations, moisture-induced dissociation, and the true commercial viability of this dual-API crystal lattice. The sustained increase in solubility from gastric to intestinal pH indicates dissolution behavior, which may contribute to the enhanced membrane permeation observed under biorelevant conditions. While solubility is expected to influence biological performance, further studies are required to establish a direct relationship between physicochemical enhancement and the observed in vitro anticancer activity.

Beyond structural characterization, a new finding of this work is that NNC substantially improves the permeability of niclosamide, which has been limited by poor solubility and bioavailability. The enhanced permeation profile and higher apparent permeability coefficients indicate that modifying the crystal lattice translates into more favorable transport across the test membrane, likely by increasing the fraction of drug available in a dissolved or readily diffusible state. To evaluate the biopharmaceutical performance of the multi-component, in vitro membrane permeation kinetics were pursued in fed-state simulated intestinal fluid, as a highly effective, biorelevant proxy for kinetic dissolution and dynamic flux. Pure niclosamide remained flat along the baseline due to its poor inherent dissolution rate and low wettability, whereas the NNC phase generated an optimized, intermediate profile yielding a distinct 3.6-fold increase in the apparent permeability coefficient Papp = 1.12 ± 0.55 × 10^−4^ cm/s. Because nafamostat mesylate is highly susceptible to rapid hydrolytic degradation in aqueous media, we utilized a specific ion-pairing reversed-phase HPLC method to separate and quantify both active backbones into distinct, sharp chromatographic peaks based on their unique retention times. This analytical approach suggests that the NNC crystal lattice coordinates a stable, balanced flux without accelerating coformer hydrolysis. Long-term thermodynamic equilibrium solubility, intrinsic dissolution rates, and formal supersaturation profiling remain important milestones for future downstream development; this preliminary kinetic screening successfully proves that the engineered crystal lattice alters the localized donor microenvironment, generating the thermodynamic drive necessary to overcome niclosamide’s typical solid-state transport barriers.

This improvement in permeability appears to have clear pharmacological consequences. NNC showed superior antiproliferative activity compared with niclosamide alone across several cancer cell lines, with particularly marked effects in lung cancer models. These results are consistent with the notion that dual-API supramolecular crystalline phase engineering can enhance cellular uptake and effective intracellular exposure, thereby amplifying the antitumor response without altering the underlying pharmacology of the individual agents. NNC exhibited lower IC_50_ values than the individual parent drugs across the tested cancer cell lines, indicating enhanced cytotoxic activity. This improvement may be associated with the increased apparent solubility and dissolution behavior of the supramolecular crystalline phase. However, because NNC is expected to dissociate in cell culture medium, the observed activity likely reflects the combined pharmacological effects of niclosamide and nafamostat.

The in vivo studies further support the translational potential of NNC by demonstrating meaningful reductions in tumor burden in the absence of obvious systemic toxicity. Interestingly, the 30 mg/kg dose achieved the greatest antitumor effect, whereas escalation to a higher dose did not yield additional benefit, suggesting a non-linear or plateaued dose–response relationship. Such behavior may reflect saturation phenomena, alterations in pharmacokinetics at higher exposures, or changes within the tumor microenvironment that limit further efficacy gains and will require dedicated mechanistic studies to elucidate. Overall, the convergence of solid-state properties, enhanced permeability, stronger in vitro anticancer activity, and robust in vivo efficacy positions NNC as a promising supramolecular crystalline phase-based therapeutic candidate and provides a compelling rationale for future work focused on detailed pharmacokinetic profiling, mechanistic investigations, and, ultimately, progression toward clinical evaluation. Although the present study demonstrates the promising physicochemical and biological performance of the niclosamide–nafamostat crystalline phase, several challenges remain before clinical translation. Future work will focus on establishing long-term formulation stability through ICH-compliant stability studies, optimizing scalable and reproducible manufacturing processes, and evaluating batch-to-batch consistency. In addition, comprehensive pharmacokinetic, bioavailability, safety, and regulatory studies will be required to confirm the therapeutic advantages and support the clinical development of this dual-active pharmaceutical cocrystal.

## 4. Materials and Methods

### 4.1. Materials

Niclosamide was purchased from Hengcheng Pharmaceuticals (Quanzhou, China). Nafamostat mesylate was obtained from Kukjeon (Hwaseong-si, Republic of Korea), and sodium bicarbonate was supplied by Emmennar (Hyderabad, India). Phosphate-buffered saline (PBS), polyvinylpyrrolidone K30 (PVP K30), sodium bicarbonate, ethanol, methanol, acetonitrile, tetrahydrofuran, 2-propanol, and N,N-dimethylformamide were purchased from Sigma-Aldrich (St. Louis, MO, USA). All reagents and solvents were of analytical grade and were used as received without further purification.

### 4.2. Niclosamide–Nafamostat Supramolecular Crystalline Phase Preparation

Nafamostat was dissolved in distilled water at room temperature under mechanical stirring at 500 rpm. Sodium carbonate was dissolved in a separate vessel, and the resulting solution was gradually added to the nafamostat solution under continuous stirring. The reaction mixture was subsequently filtered, and the filtrate was collected for further processing. In a separate vessel, niclosamide was dissolved in acetone and filtered to remove any undissolved material. The niclosamide solution was then added to the nafamostat solution, and the reaction mixture was heated to 60 °C. The combined solution was stirred at 4000 rpm for 1 h. Following the reaction, the solvent was removed under vacuum filtration. Niclosamide and nafamostat mesylate were used at a molar ratio of 1:4 during preparation of the NNC solid phase. The resulting crystalline phases were collected and dried under reduced pressure in a vacuum dryer for 24 h. Four (n = 4) independent batches were prepared under identical experimental conditions to assess the batch-to-batch reproducibility. Figure 1 shows a schematic overview of the NNC supramolecular crystalline phase process and its evaluation studies.

### 4.3. Fourier-Transform Infrared Spectroscopy

FTIR spectra were recorded using an IRSpirit FTIR spectrophotometer (Kyoto, Japan). Samples were prepared using the potassium bromide (KBr) pellet method, in which the sample was thoroughly mixed with KBr and compressed into a transparent pellet. The pellet was placed in the sample holder, and spectra were acquired at a spectral resolution of 4 cm^−1^ with 10 accumulated scans over the wavenumber range of 400–4000 cm^−1^.

### 4.4. High-Power X-Ray Diffraction

High-power X-ray diffraction (HP-XRD) data were collected using a Rigaku X-ray diffractometer (Tokyo, Japan) equipped with Cu Kα radiation operating at 40 kV and 150 mA. Measurements were performed over a 2θ range of 4–60° using a step size of 0.002°. The instrument was equipped with a rotating-anode X-ray source and a vertical wide-angle goniometer.

### 4.5. Thermal Analysis

Thermal analysis was performed using a NETZSCH STA-MS(II) instrument (Selb, Germany) under a nitrogen atmosphere with a flow rate of 40 mL/min. Samples were sealed in perforated aluminum pans and analyzed over a temperature range of 28–300 °C at a heating rate of 10 °C/min.

### 4.6. Side-by-Side Diffusion Cell Permeation Study

Membrane permeation studies were conducted using side-by-side diffusion cells (PermeGear, Hellertown, PA, USA). The experiments were conducted using a regenerated cellulose membrane with an effective diffusion area of 0.64 cm^2^ and a chamber volume of 5.0 mL on both sides. The applied dose was standardized at 2 mg of sample per donor cell. Sink conditions were strictly maintained in the receiver chamber via the addition of a 1% Kolliphor solution, which was experimentally verified to keep donor-layer accumulation well below 10% of maximum saturation throughout the 120-min run. Samples were introduced into the donor chamber and maintained at 37 °C with continuous stirring at 500 rpm. Aliquots were collected from the receiver chamber at 0, 10, 20, 30, 45, 60, 90, and 120 min. The collected samples were filtered through a polyvinylidene fluoride (PVDF) membrane filter prior to analysis by high-performance liquid chromatography (HPLC). Chromatographic analysis was performed using an Agilent 1260 HPLC system equipped with a C18 column. The mobile phase consisted of acetonitrile and an aqueous solution containing 1-sodium heptanesulfonate and acetic acid (70:30, *v*/*v*). The column temperature was maintained at 40 °C, the injection volume was 20 µL, and the flow rate was set at 1.0 mL/min.

The apparent permeability coefficient was calculated using the standard equation:

Papp = (dQ/dt)/(A.C_0_)

where (dQ/dt) represents the steady-state flux across the membrane, A is the effective diffusion area, and C_0_ is the initial donor concentration. The mass balance recovery across both chambers at 120 min was consistently represented by a minimum of three independent experiments (n = 3), reported as mean ± SD.

### 4.7. In Vitro Anticancer Assay

Human breast cancer cell lines (MCF-7 and MDA-MB), pancreatic cancer cell lines (PANC-1 and MIAPACA-2), and non-small cell lung cancer cell lines (A-549 and H-1299) were used in this study. Cells were cultured in Dulbecco’s Modified Eagle Medium supplemented with 10% fetal bovine serum and 1% antibiotic solution (Gibco) and maintained at 37 °C in a humidified atmosphere containing 5% CO_2_. Cell viability was evaluated using a Cell Counting Kit-8 (CCK-8; Abcam, Waltham, MA, USA), and IC_50_ values were determined accordingly. Cells were seeded at 2–4 × 103 cells/well in 96-well plates overnight. NNCs were treated at concentrations of 0.3, 3, 30, 300, 3000, and 30,000 nM for 48 h in triplicate. Cell viability was assessed according to the manufacturer’s protocol, and the IC_50_ was determined from the resulting dose–response curves.

### 4.8. In Vivo Anticancer Activity

Twenty female non-obese diabetic/severe combined immunodeficiency (NOD/SCID) mice (7 weeks old, weighing 20~22 g) were purchased from the Biotechnology Research Institute (Daejeon, Republic of Korea). All animal procedures were performed at the National Cancer Center in strict compliance with the Animal Protection Act and the Laboratory Animal Act of the Republic of Korea, under a protocol approved by the Institutional Animal Care and Use Committee (IACUC) on 1 June 2021 (Approval No. NCC-21-702). Mice were housed in specific pathogen-free conditions within a group of ventilated cages under a controlled 12 h light/dark cycle (Temperature 23 ± 2 °C and 45 ± 10% Humidity) with ad libitum access to sterile food and water. To establish the orthotopic pancreatic cancer model, an intraperitoneal injection of PANC-1 cells (1 × 10^6^ cells in 50 µL PBS) was performed per animal. Following tumor growth at day 50, mice were randomly assigned into four experimental groups (n = 5 per group) using a random number generator. Sample sizes were chosen based on historical facility data for the orthotopic model to ensure adequate statistical evolution. The groups included vehicle control, NNC 15 mg/kg, NNC 30 mg/kg, and NNC 60 mg/kg. Formulations were prepared in a vehicle of 97.5% PBS and 2.5% PVP K30 and administered five days per week for four weeks via intraperitoneal injection. No animals were excluded from analysis, and no formal blinding was practiced. Animal relative body weight (%) and general health were monitored daily. Humane endpoints were pre-specified as body weight loss > 20% or prolonged lethargy. There were no unexpected adverse events or premature deaths that occurred. At day 80, mice were sacrificed, and tumor burden was directly evaluated via absolute harvested tumor mass weight (g). In vivo data were evaluated using the statistical method as mentioned in the experimental section.

### 4.9. Statistical Analysis

All experimental data are expressed as the mean ± standard deviation (SD) for in vitro experiments or mean ± standard error of the mean (SEM) for in vivo cohorts. Statistical significance across multi-group data was evaluated using a one-way Analysis of Variance (ANOVA), followed by Tukey’s post hoc multiple comparisons test to apply necessary multiplicity corrections. Longitudinal or time-dependent datasets were reanalyzed utilizing a two-way repeated-measures ANOVA or mixed-effects models. All statistical computations were executed using GraphPad Prism software 8.0, and exact biological replicate numbers (n), exact *p*-values, and 95% confidence intervals (CIs) are systematically reported within the respective figure captions. A value of (*p* < 0.05) was considered statistically significant.

## 5. Conclusions

A novel NNC supramolecular crystalline phase was successfully prepared and characterized. The structural analyses are consistent with the presence of intermolecular hydrogen bonding and a distinct crystalline lattice. Thermal characterization revealed a single melting transition, indicative of a new solid-state form. The NNC exhibited enhanced membrane permeation and increased Papp relative to pure niclosamide. In vitro assays demonstrated potent anticancer activity across multiple cancer cell lines, whereas in vivo evaluation supported notable antitumor efficacy, though high-dose activity was constrained by fluid dissolution limits. Overall, this supramolecular crystalline phase platform established a solid framework for an advanced oncological drug-delivery platform for improving the therapeutic performance of niclosamide and warrants further clinical development.

## Data Availability

The original contributions of this study are provided within the article. Additional questions or requests for information may be directed to the corresponding authors.

## Supplementary Materials

The following supporting information can be downloaded at: https://www.mdpi.com/article/10.3390/ph19081277/s1, Figure S1: Overlaid Differential Scanning Calorimetry (DSC) thermograms demonstrating the batch-to-batch thermal and crystalline reproducibility of the NNC supramolecular crystalline phase. Thermograms represent independently prepared batches, recorded over a temperature range of 30 °C to 300 °C under a continuous nitrogen purge. All the independent batches demonstrate an identical and highly consistent sharp endothermic melting peak at approximately across all preparations (211.87, 212.51, 212.53, and 213.40 °C; Mean ± SD = 212.58 ± 0.63 °C). The overlapping profile confirms excellent solid-state phase uniformity and robust reproducibility of the optimized crystallization process; Figure S2: Equilibrium solubility of niclosamide and the NNC supramolecular crystalline phase in distilled water (DW) and buffer solutions at pH 1.2, 4.0, and 6.8. The supramolecular crystalline phase exhibited higher apparent solubility than niclosamide under all tested conditions, demonstrating improved aqueous solubility across a broad physiological pH range. Data are presented as mean ± SD (*n* = 3); Figure S3: Stability profiles of niclosamide, nafamostat, and NNC supramolecular crystalline phase. Samples were stored under ambient conditions (25 ± 2 °C/60 ± 5% RH). Bars represent the remaining compound percentage (%) quantified by assay. Data are shown as mean ± SD (*n* = 3); Table S1: Solid-state characterization summary and phase purity evaluation. The table details corresponding critical analytical markers across Fourier-transform infrared spectroscopy (FTIR), powder X-ray diffraction (PXRD), and differential scanning calorimetry (DSC) for pure niclosamide, nafamostat mesylate, their physical mixture (NNC-PM), and the multicomponent supramolecular crystal phase (NNC). Observed chemical vibrational shifts, unique crystal lattice reflections (2), thermal transition peaks, and phase structural descriptions support the confirmation of multicomponent crystalline phase purity distinct from a simple physical blend; Table S2: Comprehensive IC_50_ regression parameters in breast cancer cell lines. Values reflect best-fit estimates (IC_50_, LogIC_50_, R^2^) alongside their 95% confidence intervals (95% CI) across MCF-7 and MDA-MB cell lines. Open-ended limits (>300,000) denote unbounded upper constraints on nafamostat parameters due to uncompleted plateau curves; Table S3: Comprehensive IC_50_ regression parameters in pancreatic cancer cell lines. Values reflect best-fit estimates (IC_50_, LogIC_50_, R^2^) alongside their 95% confidence intervals (95% CI) across PANC-1 and MIAPACA-2 Cell lines. Open-ended limits (>300,000) denote unbounded upper constraints on nafamostat parameters due to uncompleted plateau curves; Table S4: Comprehensive IC_50_ regression parameters in Lung cancer cell lines. Values reflect best-fit estimates (IC_50_, LogIC_50_, R^2^) alongside their 95% confidence intervals (95% CI) across A-549 and H-1299 cell lines. Open-ended limits (>300,000) denote unbounded upper constraints on nafamostat parameters due to uncompleted plateau curves.
