# Supplementary material for: Supramolecular Complexation of Niclosamide and Nafamostat: Improved Physicochemical Profile and Enhanced Anticancer Effect
Source: Pharmaceuticals (Basel). 2026 Aug 13;19(8):1277. doi: 10.3390/ph19081277 (PMC13516264; doi:10.3390/ph19081277)
Supplement: Supplementary file 1 [file pharmaceuticals-19-01277-s001.zip › pharmaceuticals-4438048-supplementary.pdf]

Article

Supramolecular Complexation of Niclosamide and Nafamostat: Improved Physicochemical Profile and Enhanced Anticancer Effect

Se-Eun Byeon [1], Rengarajan Baskaran [2] and Young-Joon Park [1,2,*]

[1] *Research Center, IMDPharm Inc, 17 Daehak 4-ro , Yeongtong-gu, Suwon , Gyeonggi-do, Republic of Korea*
[2] *Ajou University, College of Pharmacy, , 206 Worldcup-ro, Yeongtong-gu, Suwon , Gyeonggi-do, Republic of Korea*
* Correspondence: parkyj64@gmail.com

Experimental Methods:

Equilibrium Solubility Studies

The equilibrium solubility of pure niclosamide and the NNC supramolecular crystalline phase was evaluated in distilled water (DW) and various physiologically relevant aqueous buffer media (pH 1.2, pH 4.0, and pH 6.8). A 20 mg of each crystalline sample was added to glass vials containing 10 mL of the respective medium to ensure the formation of saturated suspensions. The vials were securely capped and placed in a thermostatic shaking water bath maintained at 35 ± 2.0 °C and agitated at 100 rpm for 24 hrs to achieve thermodynamic equilibrium. The equilibrated samples were centrifuged at 10,000 rpm for 10 min to separate the undissolved excess solid phase. The resulting supernatant fractions were collected and passed through a 0.22-μm nylon membrane syringe filter to remove any remaining suspended microparticles. The filtered solutions were appropriately diluted, and the concentration of dissolved drug was quantified via high-performance liquid chromatography (HPLC). All solubility measurements were performed in triplicate (n = 3), and data points were reported as the mean standard deviation (± SD).

Equilibrium Solubility StudiesAmbient stability study

Ambient stability testing, accurately weighed 1 g crystalline samples of Niclosamide, Nafamostat, and NNC were stored in stability chambers under controlled conditions (25 ± 2 °C / 60 ± 5% RH) for a duration of 1 month. At the conclusion of the storage period, samples were dissolved in an appropriate solvent mixture and filtered to evaluate chemical recovery. The remaining active drug concentrations were quantified via high-performance liquid chromatography (HPLC) in triplicate (n=3), with data expressed as a percentage relative to the Initial values. This evaluation was restricted to assessing chemical recovery, and solid-state phase retention was not verified by PXRD and DSC at this time point.

Material / Phase	FTIR (cm^{-1})	PXRD (2 θ)	DSC (°C)	Solid-State Interpretation / Phase Purity
**Niclosamide**	O–H N–H bands (3500~3600); sharp amide carbonyl	13.1, 25.4, 26.8	Single sharp melting peak at 233.0 ± 0.1 °C	Pure unreacted drug crystalline framework
**Nafamostat mesylate**	Strong sulfonate (1165) and amine/guanidine	17.1, 18.4, 19.8, 24.3	Single sharp melting peak at 265.6 ± 0.2 °C	Pure unreacted codrug crystalline framework
**NNC-PM**	Superposition overlay of both starting components	Overlapping reflections of pure components (13.1, 17.1, 24.3)	Prominent melting depression peak at 231.6 ± 0.3 °C	Simple physical blend; no solid-state lattice rearrangement occurs
**NNC**	Clear shifts in the O–H/N–H and fingerprint regions	6.8, 9.1, 9.7, 13.8, 19.5, and doublet at 26.2/26.6	Broad peak at 114.8 ± 0.2 °C ; sharp melting at 213.4 ± 0.2 °C	Distinct multi-component phase; complete absence of unreacted parent material melting points

Sup. Table S1. Solid-state characterization summary and phase purity evaluation. The table details corresponding critical analytical markers across Fourier-transform infrared spectroscopy (FTIR), powder X-ray diffraction (PXRD), and differential scanning calorimetry (DSC) for pure niclosamide, nafamostat mesylate, their physical mixture (NNC-PM), and the multicomponent supramolecular crystal phase (NNC). Observed chemical vibrational shifts, unique crystal lattice reflections (2), thermal transition peaks, and phase structural descriptions support the confirmation of multicomponent crystalline phase purity distinct from a simple physical blend.

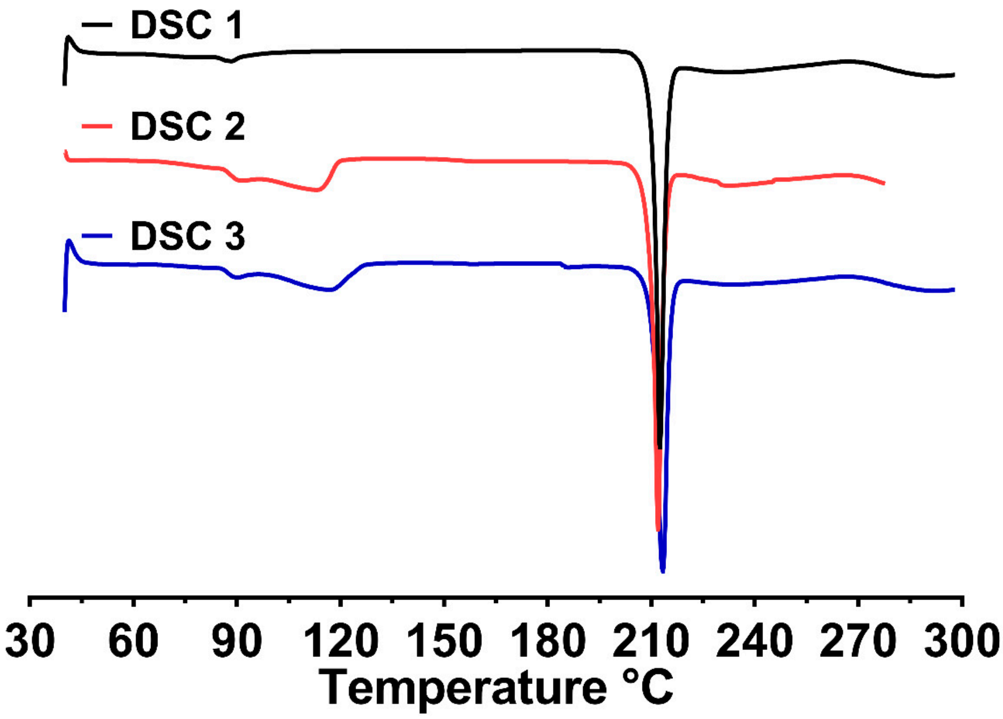

Sup. Figure S1. Overlaid Differential Scanning Calorimetry (DSC) thermograms demonstrating the batch-to-batch thermal and crystalline reproducibility of the NNC supramolecular crystalline phase. Thermograms represent independently prepared batches, recorded over a temperature range of 30 °C to 300 °C under a continuous nitrogen purge. All the independent batches demonstrate an identical and highly consistent sharp endothermic melting peak at approximately across all preparations (211.87, 212.51, 212.53, and 213.40 °C; Mean ± SD = 212.58 ± 0.63 °C). The overlapping profile confirms excellent solid-state phase uniformity and robust reproducibility of the optimized crystallization process.

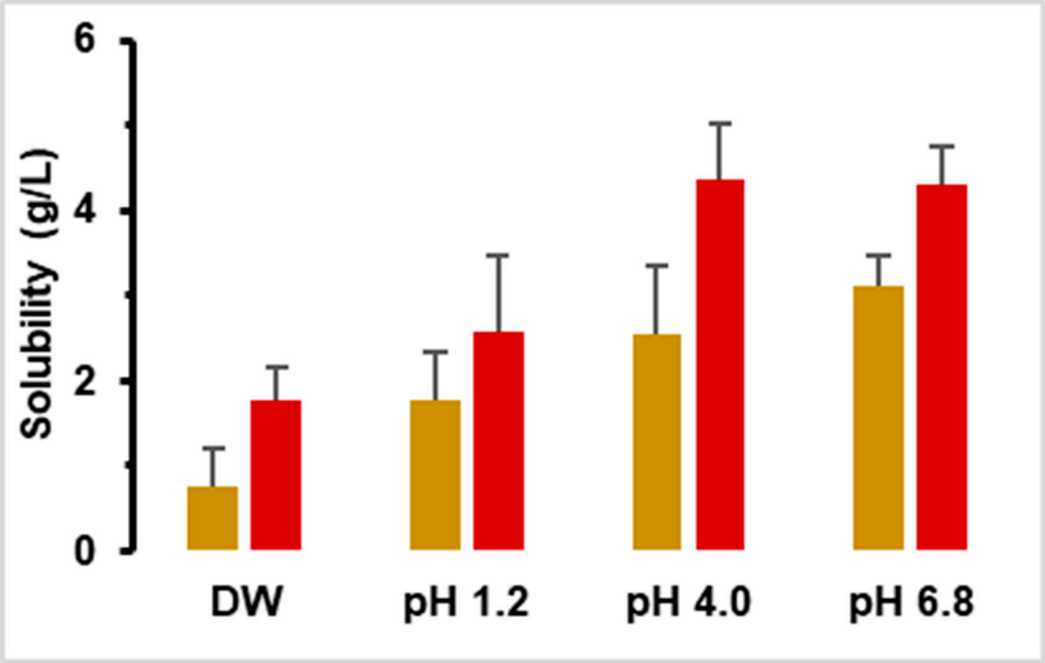

Sup. Figure S2. Equilibrium solubility of niclosamide and the NNC supramolecular crystalline phase in distilled water (DW) and buffer solutions at pH 1.2, 4.0, and 6.8. The supramolecular crystalline phase exhibited higher apparent solubility than niclosamide under all tested conditions, demonstrating improved aqueous solubility across a broad physiological pH range. Data are presented as mean ± SD (n = 3).

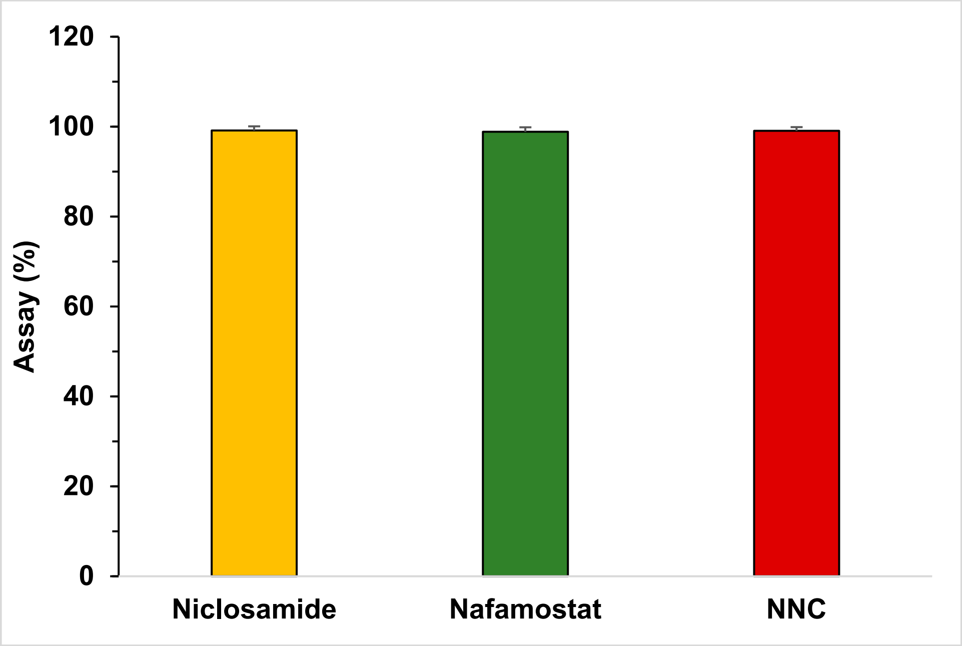

Sup. Figure S3. Stability profiles of niclosamide, nafamostat, and NNC supramolecular crystalline phase. Samples were stored under ambient conditions (25 ± 2 °C / 60 ± 5% RH). Bars represent the remaining compound percentage (%) quantified by assay. Data are shown as mean ± SD (n = 3).

		MCF-7			MDA-MB		
		IC_{50}	$LogIC_{50}$	R^2	IC_{50}	$LogIC_{50}$	R^2
**log(inhibitor) vs. response**	**NNC**	196.4	2.293	0.990	54.07	1.733	0.887
	**Niclosamide**	229.2	2.36	0.974	2042	3.31	0.875
	**Nafamostat**	7241	3.86	0.932	7163	3.855	0.766
**95% CI**	**NNC**	151.3 to 253.2	2.180 to 2.403	0.990	14.47 to 461.3	1.161 to 2.664	0.887
	**Niclosamide**	148.6 to 351.9	2.172 to 2.546	0.974	345.5 to 6748	2.538 to 3.829	0.875
	**Nafamostat**	3315 to 21802	3.520 to 4.338	0.932	1337 to >30000	3.126 to >30000	0.766

Sup. Table S2. Comprehensive IC_{50} regression parameters in breast cancer cell lines. Values reflect best-fit estimates (IC_{50}, $LogIC_{50}$, R^2) alongside their 95% confidence intervals (95% CI) across MCF-7 and MDA-MB cell lines. Open-ended limits (> 300000) denote unbounded upper constraints on nafamostat parameters due to uncompleted plateau curves.

		PANC-1			MIAPACA-2		
		IC_{50}	$LogIC_{50}$	R^2	IC_{50}	$LogIC_{50}$	R^2
**log(inhibitor) vs. response**	**NNC**	452.6	2.656	0.841	514.9	2.712	0.982
	**Niclosamide**	3462	3.539	0.837	2745	3.438	0.881
	**Nafamostat**	14122	4.15	0.906	6591	3.819	0.933
**95% CI**	**NNC**	62.76 to 14980	1.798 to 4.176	0.841	351.8 to 771.6	2.546 to 2.887	0.982
	**Niclosamide**	70.91 to 19243	1.851 to 4.284	0.837	415.7 to 9037	2.619 to 3.956	0.881
	**Nafamostat**	4781 to >30000	3.680 to >30000	0.906	3006 to 18600	3.478 to 4.270	0.933

Sup. Table S3. Comprehensive IC_{50} regression parameters in pancreatic cancer cell lines. Values reflect best-fit estimates (IC_{50}, $LogIC_{50}$, R^2) alongside their 95% confidence intervals (95% CI) across PANC-1 and MIAPACA-2 Cell lines. Open-ended limits (> 300000) denote unbounded upper constraints on nafamostat parameters due to uncompleted plateau curves.

		A-549			H-1299		
		**IC_{50}**	**$LogIC_{50}$**	**R^2**	**IC_{50}**	**$LogIC_{50}$**	**R^2**
**log(inhibitor) vs. response**	**NNC**	97.28	1.988	0.994	2.596	0.4143	0.973
	**Niclosamide**	990.9	2.996	0.938	153.2	2.185	0.982
	**Nafamostat**	25173	4.401	0.979	1642	3.215	0.880
**95% CI**	**NNC**	78.05 to 121.2	1.892 to 2.083	0.994	1.623 to 4.188	0.2103 to 0.6220	0.973
	**Niclosamide**	567.5 to 1726	2.754 to 3.237	0.938	102.4 to 224.3	2.010 to 2.351	0.982
	**Nafamostat**	12241 to 187143	4.088 to 5.272	0.979	345.1 to 5357	2.538 to 3.729	0.880

Sup. Table S4. Comprehensive IC_{50} regression parameters in Lung cancer cell lines. Values reflect best-fit estimates (IC_{50}, $LogIC_{50}$, R^2) alongside their 95% confidence intervals (95% CI) across A-549 and H-1299 cell lines. Open-ended limits (> 300000) denote unbounded upper constraints on nafamostat parameters due to uncompleted plateau curves.
